# A genetic association analysis of polymorphisms, rs2282695 and rs12373539, in the FOSB gene and papillary thyroid cancer

**DOI:** 10.3892/etm.2012.604

**Published:** 2012-06-08

**Authors:** SANG-AH HAN, JEONG-YOON SONG, SEON-YOUNG MIN, WON SEO PARK, MI-JA KIM, JOO-HO CHUNG, KEE HWAN KWON

**Affiliations:** 1 Department of Surgery, School of Medicine, Kyung Hee University, Seoul;; 2 Kohwang Medical Research Institute, School of Medicine, Kyung Hee University, Seoul;; 3 Department of Otolaryngology – Head and Neck Surgery, School of Medicine, Kyung Hee University, Seoul, Republic of Korea

**Keywords:** FOSB, polymorphism, haplotype, papillary thyroid cancer

## Abstract

The FOSB gene is involved in cell proliferation, differentiation and transformation in several tumor types. We investigated whether coding single-nucleotide polymorphisms (cSNPs) and promoter SNPs of FOSB contribute to the development of papillary thyroid cancer (PTC). We also assessed the associations between FOSB SNPs and the clinicopathological characteristics of PTC. One coding SNP (rs2282695, Ala39Ala) and one promoter SNP (rs12373539, −158) in the FOSB gene were genotyped using direct sequencing in 94 PTC patients and 213 healthy controls. Genetic data were analyzed using SNPStats, HelixTree and SNPAnalyzer. PTC patients were dichotomized and compared with respect to clinicopathological characteristics of PTC. We detected an association between PTC and cSNP (rs2282695) in FOSB [codominant model 1 (C/C vs. G/C); OR=1.75; 95% CI, 1.04–2.94; P=0.024; codominant model 2 (C/C vs. G/G): OR=2.55; 95% CI, 1.15–5.64; P=0.045; dominant model: OR=1.89; 95% CI, 1.16–3.08; P=0.010; Log-additive model: OR=1.64; 95% CI, 1.15–2.35; P=0.007]. The G allele was a risk allele in the geno-type and allele analyses of cSNP (rs2282695) in the FOSB gene (OR=1.57; 95% CI, 1.10–2.24; P=0.012). A promoter SNP (rs12373539) in FOSB was associated with cervical lymph node metastasis of PTC [codominant model 1 (G/G vs. A/G): OR=0.23; 95% CI, 0.07–0.72; P=0.016; codominant model 2 (G/G vs. A/A): OR=0.21; 95% CI, 0.02–1.96; P=0.0.05; dominant model: OR=0.22; 95% CI, 0.08–0.66; P=0.004; overdominant model: OR=0.27; 95% CI, 0.09–0.84; P=0.02; log-additive model: OR=0.31; 95% CI, 0.12–0.78; P=0.006]. The A allele was a protective allele in the genotype and allele analyses of SNP (rs12373539) in the FOSB gene promoter (OR=0.34; 95% CI, 0.14–0.83; P=0.017). Variation in a FOSB cSNP (rs2282695) may be associated with risk of PTC. The FOSB promoter SNP (rs12373539) may be associated with lymph node metastasis of PTC.

## Introduction

sPapillary thyroid carcinoma (PTC) is the most common endocrine malignancy, accounting for more than 90% of all endocrine malignancies. The incidence of thyroid cancer has increased more than 2-fold in the US during the past three decades, mainly due to increases in papillary thyroid cancer (PTC), which is the predominant type of malignant thyroid tumor (1). Due to a steady increase in the thyroid cancer incidence in Korea, thyroid cancer has become the most common cancer in Korean women (2).

The development of thyroid cancer is strongly determined by individual genetic background. A genetic predisposition for PTC has been suggested by case-control studies showing a 3- to 8-fold increase in risk in first-degree relatives, which is one of the highest such risks of any cancer type (3,4). Despite unequivocal evidence of heritability, large families displaying Mendelian inheritance of PTC are rare, and no predisposing genetic factors have been convincingly described (5,6). In this regard, it is expected that sporadic thyroid cancer is the result of multiple low- to moderate-penetrance genes interacting with each other and with the environment, thus modulating individual susceptibility. Screening and description of single-nucleotide polymorphisms (SNPs) in certain genes that are involved in carcinogenesis have been the main approaches to characterizing inter-individual differences in genetic predisposition for cancer development.

FBJ murine osteosarcoma viral oncogene homolog B, also known as FOSB (in humans) or FosB (in other species), is a protein that is encoded by the FOSB gene in humans (7–9). The Fos gene family consists of 4 members: FOS, FOSB, FOSL1 and FOSL2. These genes encode leucine zipper proteins that dimerize with proteins of the JUN family, thereby forming the transcription factor complex AP-1. AP-1 regulates gene expression in response to a variety of stimuli, including cytokines, growth factors, stress, and bacterial and viral infections (10). AP-1 in turn controls a number of cellular processes including differentiation, proliferation, and apoptosis (11). Therefore, the FOS proteins have been implicated as regulators of cell proliferation, differentiation, apoptosis and carcinogenesis.

In this study, we investigated whether SNPs in the FOSB gene contribute to the development of PTC. We also assessed the relationships between the FOSB gene and the clinicopathological characteristics of PTC.

## Materials and methods

### Subjects and controls

We recruited 94 patients with PTC and 213 control patients at Kyung Hee University Medical Center, Seoul, Republic of Korea. The diagnosis of PTC and the presence of cervical regional lymph node metastasis were confirmed by pathological examination. The specimens that were diagnosed as follicular variants, diffuse sclerosing and tall cell variants were excluded. None of the controls were diagnosed with cancer or thyroid disease at the time of enrollment. The male-to-female ratio of the included patients was 27:67 and the mean age was 53.2±12.0 years. The control group of 213 healthy adults (mean age, 55.4±6.0 years) included 108 males and 105 females. This study was approved by the Institutional Review Board of the Medical Research Institute, Kyung Hee University Medical Center. Written informed consent was directly obtained from all subjects.

### Patient subgroups

To determine the nature of the relationship between FOSB SNPs and the clinicopathological characteristics of PTC, patients were divided into subgroups according to size (<1 or ≥1 cm), number (unifocality or multifocality) and location of cancers (one or both lobes). In addition, PTC patients were also subgrouped into extrathyroidal invasion (+) and (-) groups based on pathological findings. Finally, PTC patients were further subgrouped into lymph node metastasis (+) and (-) groups to evaluate the contribution of FOSB SNPs to cancer metastasis. The demographic characteristics of PTC patients are summarized in Table I; small differences in subgroup numbers were caused by loss of some clinical data.

### SNP selection and genotyping

We searched the oding SNPs (cSNPs) of the FOSB gene. Information related to the SNPs was obtained from the National Center of Biotechnology Information (NCBI) SNP database (www.ncbi.nlm.nih.gov/ SNP, dbSNP BUILD 135). Among the SNPs of FOSB, SNPs with heterozygosity <0.05 or unknown and monogenotype were excluded. For genetic analysis, we selected rs2282695 (coding sequence, average heterozygosity 0.479) and rs12373539 (promoter sequence, average heterozygosity 0.345). Genomic DNAs were extracted from blood samples collected in EDTA tubes using the Roche DNA Extraction kit (Roche, Indianapolis, IN, USA). SNP genotyping was conducted by direct sequencing. Polymerase chain reaction (PCR) was performed using specific primers for the FOSB SNPs that were selected for analysis (Table II). PCR products were sequenced using an ABI PRISM 3730XL analyzer (PE Applied Biosystems, Foster City, CA, USA), and sequence data were analyzed using SeqManII software (DNAStar, Madison, WI, USA).

### Statistical analysis

Continuous variables are presented as the mean ± SD and were analyzed by independent t-test and Chi-square test. Hardy-Weinberg equilibrium (HWE) was assessed using SNPStats software (http://bioinfo.iconcologia.net/index.php?module=Snpstats) in patients and controls, and adjusted for gender and gender. We used HelixTree (Golden Helix Inc., Bozeman, MT, USA) and SNPAnalyzer (ISTECH Inc., Goyang, Republic of Korea) to analyze genetic data. Multiple logistic regression models (codominant, dominant, and recessive) were employed to obtain odds ratios (ORs), 95% confidence intervals (CIs) and P-values. All data analysis was performed using SPSS 18.0 software (SPSS Inc., Chicago, IL, USA). Statistical significance was set at P<0.05.

## Results

We detected a significant difference between PTC patients and controls with respect to gene allele frequencies of rs2282695. The frequency of the G allele of rs2282695 was greater in the PTC patients than in the controls (36.7 vs. 26.9%, respectively; P=0.012) (Table III). The G allele was a risk allele in the geno-type and allele analyses of cSNP (rs2282695) in the FOSB gene (OR=1.57, 95% CI, 1.10–2.24, P=0.012). However, no significant difference was observed in rs12373539 gene allele frequencies between the two groups (Table III).

The genotypic distributions of the two SNPs examined in this study were in Hardy-Weinberg equilibrium (P>0.05, data not shown). In our analyses of genotype data collected from 94 PTC patients and 213 controls, the synonymous cSNP rs2282695 of FOSB was associated with development of PTC [codominant model 1 (C/C vs. G/C): OR=1.75; 95% CI, 1.04–2.94; P=0.024; codominant model 2 (C/C vs. G/G): OR=2.55; 95% CI, 1.15–5.64; P=0.045; dominant model (C/C vs. G/C+G/G): OR=1.89; 95% CI, 1.16–3.08; P=0.010]. However, the promoter SNP rs12373539 of FOSB was not associated with development of PTC (Table III).

When we assessed the genetic relationships between SNPs and subgroups of PTC patients according to cervical lymph node metastasis, FOSB SNP (rs2282695) was not associated with cervical lymph node metastasis (data not shown). However, FOSB SNP (rs12373539) was associated with cervical lymph node metastasis (Table IV) [codominant model 1 (G/G vs. A/G): OR=0.23, 95% CI 0.07–0.72, P=0.016; codominant model 2 (G/G vs. A/A): OR=0.21, 95% CI 0.02–1.96, P=0.05; dominant model: OR=0.22, 95% CI 0.08–0.66, P=0.004; overdominant model: OR=0.27, 95% CI 0.09–0.84, P=0.02; log-additive model: OR=0.31, 95% CI 0.12–0.78, P=0.006]. The A allele was a protective allele in the genotype and allele analyses of SNP (rs12373539) in the FOSB gene promoter (OR=0.34, 95% CI 0.14–0.83, P=0.017). FOSB SNPs rs2282695 and rs12373539 were not associated with tumor size, bilaterality, angiolymphatic invasion, extrathyroidal invasion or multifocality.

## Discussion

Associations between SNPs and the risk of differentiated thyroid cancer have recently been investigated in studies focused on polymorphisms of genes that affect physiological pathways such as DNA repair, cell-cycle control, kinase-dependent signaling, endogenous or exogenous metabolisms and apoptosis (12–15). However, polymorphisms (rs2282695 and rs12373539) of the FOSB gene have not been reported to be associated with susceptibility to cancers. In this study, we showed for the first time that a FOSB SNP (rs2282695) is associated with PTC. We investigated the associations between FOSB SNPs (rs2282695 and rs12373539) and PTC. We also assessed the correlation between FOSB SNPs (rs2282695 and rs12373539) and the clinicopathological characteristics of PTC. Here, we found that: i) synonymous SNP (rs2282695, Ala39Ala) of FOSB is associated with the development of PTC; and ii) promoter SNP (rs12373539, −158) of FOSB is associated with the nodal metastasis of PTC. The SNPs rs2282695 and rs12373539 were not associated with tumor size, bilaterality, angiolymphatic invasion, extrathyroidal invasion or multifocality. Although PTC generally has a favorable prognosis following appropriate treatment, nodal metastasis of PTC is an indication for complete thyroidectomy and post-operative radioactive iodine ablation. While the long-term benefits of routine central neck dissection on recurrence and survival remain questionable, the identification of a predictor of nodal metastasis is clinically important (16).

FosB is an acidic protein of 338 amino acids that shares structural similarities with the prototype of the Fos family, c-Fos, namely, a proline-rich basic DNA binding region, a leucine zipper required for dimer formation, and a C-terminal transactivation domain (17). The human FosB gene is located on chromosome 19q13 and is composed of four exons. The 5.1-kb transcript is subject to alternative splicing: in addition to the long form, a shorter mRNA is generated by removal of a 140-bp fragment within exon 4, leading to premature termination of translation and generation of a smaller protein of 237 amino acids, called FosB2 or FosBS (18), which lacks the transactivation domain. Both proteins have similar binding properties to c-Jun, but FosB2 lacks some transforming and trans-activating properties of FosB (18,19). From experimental studies, the FOS proteins have been implicated as regulators of cell proliferation, differentiation, apoptosis, and carcinogenesis. The composite transcription factor ‘activating protein 1’ (AP-1) is a homodimeric or heterodimeric DNA-binding protein composed of either two Jun family proteins (c-Jun, JunB, JunD) or one Jun and one Fos family protein (c-Fos, FosB, Fra-1, Fra-2) (20–22), yielding 18 possible AP-1 dimers. This number is further increased by possible dimerization with other basic region-leucine zipper (bZIP) proteins such as activation transcription factors (ATF family), Jun dimerization partners (JDP proteins) and Maf proteins (23,24). The activated AP-1 dimer binds to specific DNA sequences in the regulatory regions of mitogen-responsive genes in the promoter regions of target genes (25), several of which are involved in cellular processes such as proliferation (cyclin D1, Rb, p16) or tumor invasion (most matrix-metalloproteinases, uPA, PAI-1) (23).

The role of AP-1 proteins in tumors of the thyroid gland was first investigated in the rat cell system (26). In the cited study, neoplastic transformation was associated with a drastic increase in AP-1 activity, which reflects multiple compositional changes. The strongest effect is represented by the marked junB and fra-1 gene induction. The inhibition of Fra-1 protein synthesis by stable transfection with a fra-1 antisense RNA vector significantly reduces the malignant phenotype of the transformed thyroid cells, indicating a pivotal role for the fra-1 gene product in the process of cellular transformation. Changes in c-Fos and Fra-2 expression were not observed in this experimental system. Reduced c-Fos expression in malignant papillary carcinomas in comparison with benign human thyroid tissue was observed by Liu *et al* (27). Fra-1 expression was examined using immunohistochemistry in 186 thyroid tissue samples (28). Fra-1 protein and mRNA were undetectable in normal tissues, but abundant in 100% of the carcinoma samples. In adenomas (88%) and goiters (36%), moderate Fra-1 expression was detected in certain cases. Fra-1 activation appears to be an early event in thyroid carcinogenesis (27,29). In contrast to the bulk of data on the function of c-Fos and Fra-1, far less is known about the role of FosB. Other than certain regulatory properties in the hypothalamus and cortex, the specific function of FosB in human tissue has not been identified, and its role in carcinogenesis is unknown. The reported effect of FosB on cell proliferation is opposite to that of c-Fos, Fra-1 and Fra-2. Progression of mammary carcinomas involves downregulation of FosB as well as upregulation and phosphorylation of Fra-1 and Fra-2 in an *in vivo* system (25). It is possible that common variation in the FOSB DNA sequence is associated with variation in AP-1 activity. This may reflect the possibility that cell proliferation, tumor invasion and further carcinogenic events are, to some extent, a continuum.

To determine whether the promoter SNP affects transcription factors, we used the online program AliBaba 2.1 (http://www.gene-regulation.com/pub/programs/alibaba2). At the sites of the FOSB SNP (rs12373539), the A-containing sequences bind with C/EBPdel, Oct-1, and Oct-5 transcription factors, and the G-containing sequences bind with Sp1, Oct-1, and N-Oct-4 transcription factors. Assuming that transcription factor binding varies with promoter SNPs, this promoter SNP may influence gene and protein expression of FOSB SNP (rs12373539).

This study had several limitations. The control group did not undergo thyroid ultrasonography to exclude potentially undetected thyroid cancers. Considering the incidence of thyroid cancer (0.5–10/100,000 person), this limitation does not greatly impact on this study. Small sample size was another limitation. To confirm our results, an additional study with a larger sample size should be conducted. Another limitation is the absence of tumor tissue analysis. Genetic changes of FOSB should be examined in cell lines, and immunohistochemical studies with specimen tissues are required to determine biological effects.

In conclusion, our results indicate that a synonymous SNP (rs2282695, Ala39Ala) of the FOSB gene is associated with the development of PTC, and that a promoter SNP (rs12373539, −158) of the FOSB gene may be associated with the nodal metastasis of PTC. The G allele of the FOSB SNP (rs2282695, Ala39Ala) may be a risk factor for development of PTC in the Korean population.

## Figures and Tables

**Table I:** Demographic characteristics of the study participants.

Variable	Patients with papillary thyroid cancer	Controls
Gender (M:F)	27:67	108:105
Average age (mean ± SD; years)	53.2±12.0	55.4±6.0
Cancer size, n (%)		
<1 cm	44 (47.8)	
≥1 cm	48 (52.2)	
Number of cancers, n (%)		
Unifocality	61 (67.0)	
Multifocality	30 (33.0)	
Location of cancers, n (%)		
One lobe	65 (71.4)	
Both lobes	26 (28.6)	
Extrathyroidal invasion, n (%)		
Absent	44 (48.4)	
Present	47 (51.6)	
Cervical lymph node metastasis, n (%)		
Absent	62 (80.5)	
Present	25 (19.5)	

**Table II:** Primer sequences for the FOSB SNPs analyzed in this study.

Gene	SNP		Sequence (5′-3′)	Product size (bp)
*FOSB*	rs2282695 Ala39Ala	Forward	GACTTGCACCTTACTTCCCCAAC	328
		Reverse	TCTCAGATCTAGGGTTCTGATG	
*FOSB*	rs12373539, promoter −158	Forward	TGACGTCATTGCTAGGATACCA	350
		Reverse	GGCCGTAGCTCTGAGTCTTATG	

**Table III:** Genotype and allele frequencies of SNPs of FOSB gene in PTC and control subjects.

SNP	Type	Control n (%)	PTC n (%)	Model	OR (95% CI)	P-value	Bonferroni-corrected P-value
rs2282695 Ala39Ala							
Genotype	C/C	142 (54.6)	38 (40.4)	Codominant 1^a^	1.75 (1.04–2.94)	**0.024**	0.07
	G/C	96 (36.9)	43 (45.7)	Codominant 2^b^	2.55 (1.15–5.64)	**0.045**	0.14
	G/G	22 (8.5)	13 (13.8)	Dominant	1.89 (1.16–3.08)	**0.010**	0.03
				Recessive	1.96 (0.93–4.14)	0.09	0.27
				Overdominant	1.47 (0.90–2.39)	0.12	0.36
				Log-additive	1.64 (1.15–2.35)	**0.007**	**0.021**
Allele	C	380 (73.1)	119 (63.3)		1		
	G	140 (26.9)	69 (36.7)		1.57 (1.10–2.24)	**0.012**	**0.036**
rs12373539 (−158)							
Genotype	G/G	115 (44.2)	49 (52.1)	Codominant 1^c^	0.75 (0.45–1.24)	0.43	1.00
	A/G	120 (46.1)	38 (40.4)	Codominant 2^d^	0.66 (0.26–1.66)	0.36	1.00
	A/A	25 (9.6)	7 (7.5)	Dominant	0.73 (0.45–1.18)	0.20	0.60
				Recessive	0.76 (0.31–1.85)	0.54	1.00
				Overdominant	0.79 (0.49–1.29)	0.35	1.00
				Log-additive	0.78 (0.53–1.15)	0.21	0.63
Allele	G	350 (67.3)	136 (72.3)		1		
	A	170 (32.7)	52 (27.7)		0.79 (0.55–1.14)	0.20	0.60

a Codominant 1, C/C vs. G/C;

b Codominant 2, C/C vs. G/G;

c Codominant 1, G/G vs. A/G;

d Codominant 2, G/G vs. A/A. Bold type indicates p<0.05.

**Table IV:** Genotype and allele frequencies of rs123783539 of the FOSB gene in PTC patients with LN metastasis.

SNP	Type	LN meta (con) n (%)	LN meta (PTC) n (%)	Model	OR (95% CI)	P-value	Fisher exact P-value	Bonferroni-corrected P-value
rs12373539(−158)								
Genotype	G/G	28 (45.2)	19 (76.0)	Codominant 1^a^	0.23 (0.07–0.72)	**0.016**	**0.025**	0.08
	A/G	28 (45.2)	5 (20.0)	Codominant 2^b^	0.21 (0.02–1.96)	0.05	0.24	0.72
	A/A	6 (9.7)	1 (4.0)	Dominant	0.22 (0.08–0.66)	**0.004**		**0.012**
				Recessive	0.36 (0.04–3.24)	0.32	0.67	1.00
				Overdominant	0.27 (0.09–0.84)	**0.02**	**0.031**	0.09
				Log-additive	0.31 (0.12–0.78)	**0.006**		**0.018**
Allele	G	84 (67.7)	43 (86.0)		1			
	A	40 (32.3)	7 (14.0)		0.34 (0.14–0.83)	**0.017**		0.05

a Codominant 1, G/G vs. A/G;

b Codominant 2, G/G vs. A/A. Bold type indicates p<0.05.
